# Comparing Laparoscopic Versus Open Repair of Recurrent Incisional Hernia: An Updated Systematic Review and Meta‐Analysis of Randomized Controlled Studies on Recurrence and Complications

**DOI:** 10.1002/wjs.70272

**Published:** 2026-04-03

**Authors:** Ahmad R. Al‐Qudimat, Ahmad Zarour, Raghad Al‐Taweel, Amal I. Al‐Awadat, Hiba Bawadi

**Affiliations:** ^1^ QU Health Qatar University Doha Qatar; ^2^ Surgical Research Section Surgery Department Hamad Medical Corporation Doha Qatar; ^3^ Acute Care Surgery Surgery Department Hamad Medical Corporation Doha Qatar; ^4^ Medicine Collge, QU‐Health Qatar University Doha Qatar; ^5^ Biomedical Sciences Department QU Health Qatar University Doha Qatar; ^6^ Medicine College University of Jordan Amman Jordan; ^7^ Department of Nutrition Sciences College of Health Science QU‐Health Qatar University Doha Qatar

**Keywords:** clinical outcomes, incisional hernia, laparoscopic, open surgery, recurrence rate

## Abstract

**Background:**

Incisional hernia is the most common postoperative complication of abdominal wall surgery that significantly increases morbidity. We aimed to evaluate recurrence rates and perioperative outcomes associated with Laparoscopic and open repair for Incisional hernia recurrence.

**Methods:**

A comprehensive and systematic literature search was conducted across PubMed, Embase, CINAHL Ultimate, Medline, Scopus, and the Cochrane Controlled Trials Register. Studies published between July 2013, and November 2024 were screened for inclusion. The data extracted included recurrence rates, surgical complications, bowel injury, and outcomes. Pooled risk ratios (RR) were calculated to compare the outcomes of Laparoscopic repair and Open repair. Statistical analysis was performed using STATA V17 using a random‐effects model.

**Results:**

A total of 15 randomized controlled trials (RCTs), encompassing 1502 patients from nine countries, met the eligibility criteria. The analysis revealed no significant difference in recurrence rates between LR and OR. However, LR was associated with a significantly lower likelihood of wound drainage (RR = 0.07, 95% CI: 0.04–0.15) and a reduced risk of postoperative infection (RR = 0.31, 95% CI: 0.17–0.55) compared to OR. Conversely, the risk of bowel injury (RR = 2.80, 95% CI: 1.15–6.80, *p* = 0.02), indicating that patients undergoing LR were nearly three times more likely to experience bowel injury than those undergoing OR.

**Conclusion:**

Laparoscopic repair of incisional hernias offers several advantages over open repair, including lower rates of wound infection. However, it is associated with a higher risk of bowel injury, necessitating careful patient selection and surgical expertise.

## Graphical abstract

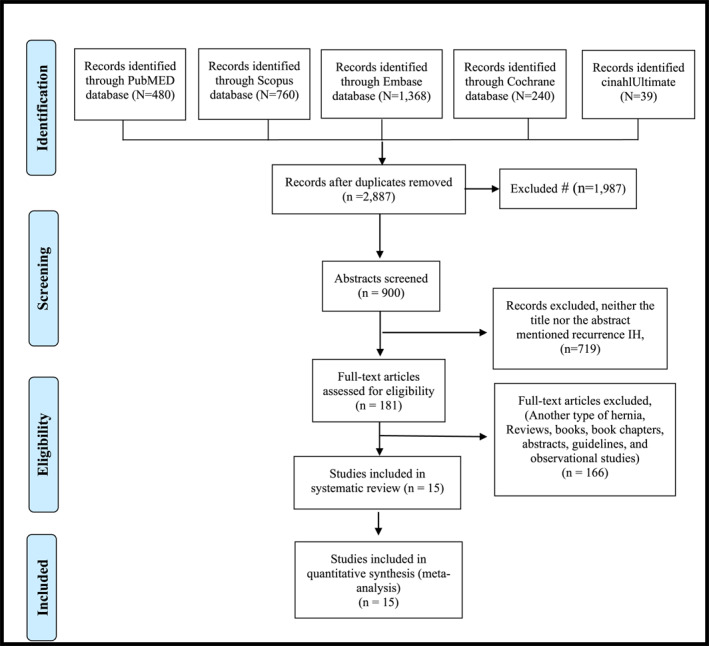

## 1 Introduction

Incisional hernia (IH) is a type of ventral hernia that occurs at or near the site of a previous surgical incision through the abdominal wall. It results from a defect or weakness in the abdominal wall closure, allowing intra‐abdominal tissues such as omentum, fat, or organs to protrude or bulge beneath or through the scar [1, 2]. IH patients have several complaints, such as pain, intestinal obstruction, strangulation, and ischemia of the herniated contents, which impact their quality of life [3, 4].

Despite improvements in surgical techniques, IH remains associated with notable morbidity and mortality, underscoring the need for effective management strategies [5, 6]. Several risk factors leading to occur IH include wound infection, male sex, obesity, abdominal distension, underlying medical conditions, and suboptimal surgical closure [7, 8]. It's important to consider these factors in preoperative planning to minimize the risk of IH development. Currently, surgical intervention is the only definitive treatment for IH, with three main approaches: open repair (OR), laparoscopic repair (LR), and robot‐assisted repair (RR). OR can be performed with or without mesh, where mesh‐based techniques are strongly recommended by the European Hernia Society (EHS) for midline incisional hernia repair, as they significantly reduce recurrence rates compared to suture repair [9, 10, 11]. On the other hand, LP involves the use of mesh minimally invasive, providing advantages consisting of decreased postoperative pain and shorter hospital stays [12, 13]. Both approaches have their advantages and limitations, and the choice of technique often depends on patient‐specific factors, hernia characteristics, and surgeon expertise.

Over the last 20 years, there have been many improvements in hernia repair. IH repairs using primary suturing have shown recurrence rates between 12% and 54% [1, 14], Meanwhile, mesh repairs, which are now standard of care per EHS recommendations, demonstrate much lower recurrence rates, though they may still reach up to 36% [2, 5, 11]. There is still a limited consensus on the comparative efficacy of LR and OR, with ongoing debate about which approach is superior. The 2005 guidelines of the Society for Surgery of the Alimentary Tract (SSAT) recommended that small hernias (< 3 cm) could often be repaired without mesh, while larger or more complex hernias, particularly those requiring component separation, were better suited for open repair. In contrast, the 2023 European Hernia Society (EHS) guidelines now advocate a more individualized, evidence‐based approach recommending routine mesh reinforcement for midline IH repair to minimize recurrence, and emphasizing that the choice between open, laparoscopic, or robotic repair should be guided by hernia size, defect location, patient comorbidities, and surgeon expertise [11, 15]. The success of hernia repair should align with these guidelines while also considering individual patient factors to determine the most appropriate surgical approach. Moreover, current evidence evaluates repair techniques based on various outcomes, including recurrence rates, cost‐effectiveness, postoperative complications, and long‐term patient prognosis [11]. Planning carefully before surgery is an essential key to optimizing surgical outcomes.

Despite the growing body of evidence on IH recurrence, significant gaps remain in the literature. Previous systematic reviews have analyzed recurrence rates using data from only five studies [16]. However, this systematic review updates the existing body of research by incorporating 10 additional studies, expanding the total to 15. This systematic review and meta‐analysis aim to comprehensively evaluate the recurrence rates and contributing factors associated with IH repair.

## 2 Methods

**FIGURE 1:**
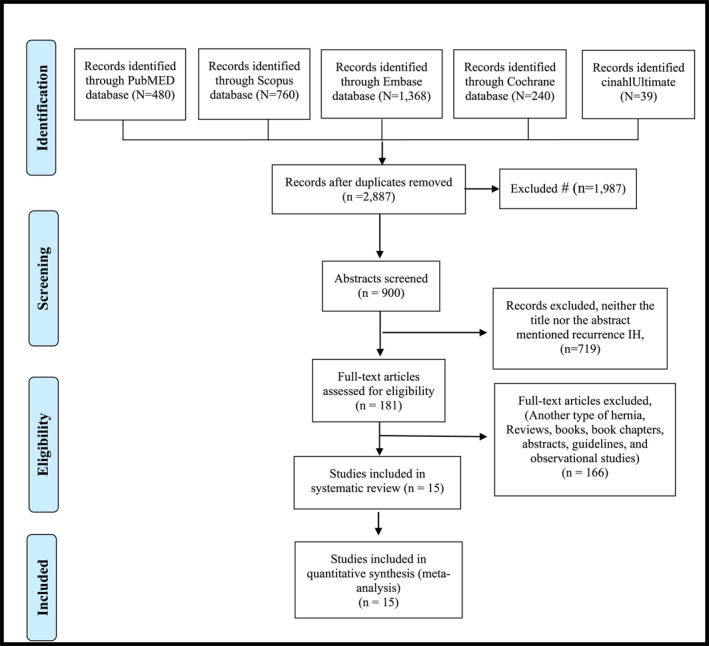
Preferred reporting items for systematic reviews and meta‐analyses flowchart.

We performed the current systematic review and meta‐analysis according to the Preferred Reporting Items for Systematic Reviews and Meta‐Analyses (PRISMA) guidelines [17], review was not registered (Figure 1).

### 2.1 Search Strategy

A comprehensive and systematic search was conducted to identify relevant studies about incisional hernia. We systematically searched through PubMed, Embase, CINAHL Ultimate, Medline, Scopus, and Cochrane controlled trials register. The search of this work covered studies published from database inception up to November 2024 and was conducted between July 2023 and November 2024 as part of an update to a previously published systematic review [16]. The search terms included “Incisional hernia,” AND “laparoscopic” OR “laparoscopic surgery,” AND “Open” OR “Open surgery.” All retrieved article titles and abstracts were initially screened, and those meeting the inclusion criteria were carefully assessed before being included in the final analysis.

### 2.2 Eligibility Criteria & Study Selection

We determined study eligibility using the PICO framework (P  =  Patient population, I  =  Intervention, C  =  Comparator, and O  =  Outcomes). As follows: Population (P): Adult patients diagnosed with incisional hernia who underwent surgical repair. Intervention (I): Laparoscopic repair using mesh for incisional hernia. Comparator (C): Open repair with or without mesh. Outcomes (O): Primary outcome was recurrence rate; secondary outcomes included postoperative complications, bowel injury, length of hospital stay, operative time, and postoperative pain. The studies that were included in the analysis were selected based on the following criteria: (1) Adult patient underwent incisional hernia repair only (hernia mesh repair), (2) open and laparoscopic repair, (3) Randomized controlled trial, (4) operated recurrence, and (5) English language only. The total number of searches that were retrieved from each database was recorded. The exclusion criteria were as follows: (1) duplicate reports (including identical patient information), (2) non‐incisional hernias or pediatric studies, (3) non‐comparative studies, (4) Cohort (prospective, and retrospective), case reports, reviews, abstracts, or unpublished data, and (5) books, guidelines, or protocols were excluded.

According to the inclusion criteria, two authors (ARA, RA) screened and evaluated the relevant articles that were found in each database. Where opinions differed, discussions were held with the third reviewer (AZ) until an agreement was reached.

### 2.3 Data Extraction

Two reviewers (ARA, RA) independently used a specific format to extract data from the included studies. Data elements included the (1) study characteristics (first author's name, publication year, recruitment period, study location, sample size), (2) Patient characteristics (age mean or median, sex ratio, BMI, defect size, mesh type, fixation method, surgical technique, follow‐up period), (3) Primary outcome (recurrence rate), and secondary outcomes (infection rate, wound drainage, bowel injury, bowel injury, hospital stay, seroma formation, mesh infection or rejection, operation duration, readmission, and reoperation if available).

### 2.4 Assessment of Bias

**FIGURE 2:**
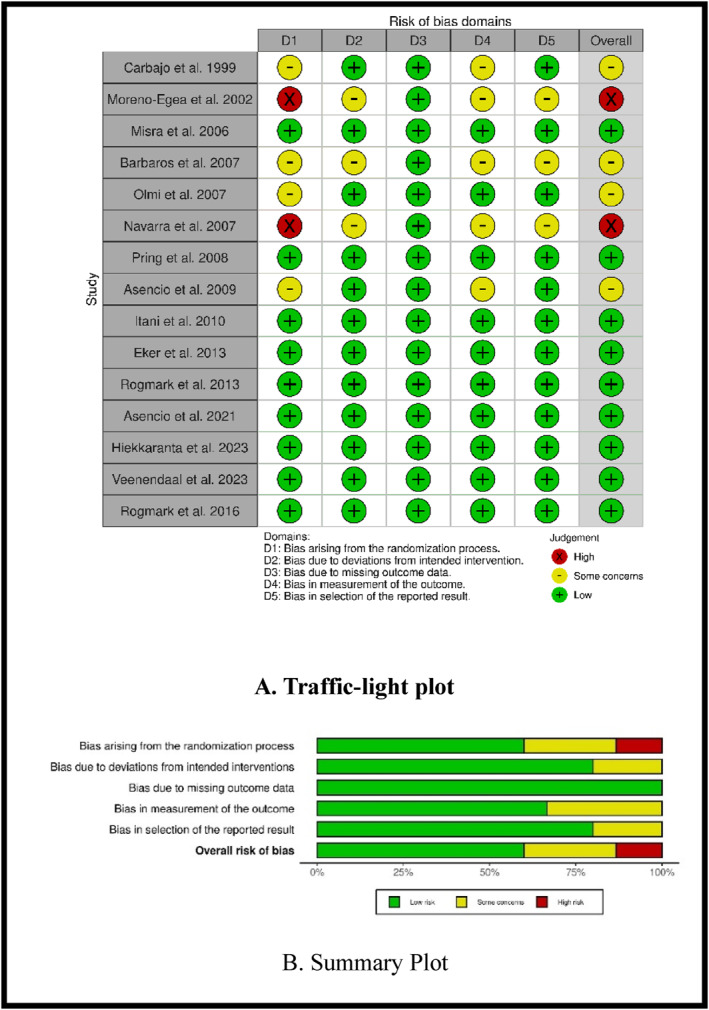
Risk assessment Bias ROB 2.

The risk of bias for each included RCT was assessed using the Cochrane Risk of Bias Tool (RoB 2) [18]. This tool evaluates RCTs across five domains: (1) the randomization process, (2) deviations from the intended interventions, (3) missing outcome data, (4) measurement of the outcome, and (5) selection of the reported results. Each domain was judged as having a low risk of bias, some concerns, or a high risk of bias, based on the study's adherence to methodological rigor. Two independent reviewers conducted the assessments, and any discrepancies were resolved through discussion or consultation with a third reviewer. Studies with a low risk of bias across all domains were considered to have the highest methodological quality, while those with some concerns or high risk of bias were flagged for potential limitations in their design or reporting. The overall risk of bias for each study was determined by the highest level of bias identified in any domain. This rigorous assessment ensures transparency and reliability in evaluating the quality of the evidence included in this systematic review (Figure 2).

### 2.5 Statistical Analysis

To compare the risk of recurrence of IH, risk ratios (RR) with 95% confidence interval, and a random‐effects meta‐analysis were used. We assessed the presence of heterogeneity across studies using the *I* ^2^ statistic, which measures the percentage of variability that can be attributable to between‐study differences. Little heterogeneity, moderate heterogeneity, and high heterogeneity are all denoted by *I* ^2^ values of 25%, 25%–75%, and > 75%, respectively [19], and Cochran Q test was used to assess the presence of heterogeneity between studies, and a *p*‐value of less than 0.05 was taken as proof of statistically significant heterogeneity. We used the Begg and Egger test and funnel plots to depict potential asymmetries between studies to evaluate the possibility of a small‐study effect or publication bias [20]. *p*‐values that were < 0.05 were considered statistically significant. All statistical tests were used Stata v.17.

## 3 Results

### 3.1 Study Characteristics

Initially, 2887 records were identified across five databases: PubMed (480), Scopus (760), Embase (1,368), CinahlUltimate (39), and the Cochrane Library (240). After removing 900 duplicates, a total of 1987 unique records remained for screening. Following the evaluation of titles and abstracts, 719 studies were excluded due to irrelevance (51), the inclusion of other hernia patient types (137), or being non‐comparative sources such as reviews, books, book chapters, abstracts, guidelines, and observational studies (531).

A total of 181 full‐text articles were assessed for eligibility. Of these, 166 studies were excluded for not involving patients who underwent LR or OR surgery (113 studies) or for not being comparative studies (53 studies). Ultimately, 15 RCTs were included in this systematic review (Figure 1).

### 3.2 Assessment of Bias

The risk of bias assessment using the Cochrane RoB 2 tool revealed that the majority of the included RCTs (9 out of 15) were judged to have a low risk of bias overall. These studies demonstrated robust randomization processes, minimal deviations from intended interventions, low rates of missing outcome data, and appropriate measurement and reporting of outcomes. However, two studies raised concerns [21, 22] were judged to have a high risk of bias, primarily due to issues with the randomization process and potential deviations from intended interventions. Additionally, Carbajo et al. (1999), Barbaros et al. (2007), Olmi et al. (2007), and Asencio et al. (2009) [12, 23, 24, 25] were assessed as having some concerns, mainly related to the randomization process and measurement of outcomes. These concerns highlight the need for cautious interpretation of results from these studies (Figure 2).

### 3.3 Patients' Characteristics

A total of 15 RCTs, including 1502 patients from nine different countries, were analyzed. The studies were conducted in Spain (4 studies), Italy (2), Sweden (2), Netherlands (2), USA (1), Australia (1), India (1), Turkey (1), and Finland (1). These studies were published between 1999 and 2023, with all 15 studies being RCTs.

The total number of patients who underwent LR was 691, while OR included 740 patients. The mean age for LR patients was 57.6 years, whereas for OR patients, it was 58.3 years. The mean BMI was 29.3 kg/m^2^ for LR patients and 29.5 kg/m^2^ for OR patients. Additionally, the average follow‐up duration was 36.2 months for LR and 28.5 months for OR, Follow‐up durations varied widely across studies and were not normally distributed. In terms of hernia overlap (mesh overlap distance), reported data showed a mean of 3–10 cm for LR and 3–10.5 cm for OR, depending on defect size and surgical technique. Regarding mesh fixation, most studies used sutures alone or a combination of sutures and staples, while tackers were rarely used. Both LR and OR approaches demonstrated similar fixation preferences, reflecting contemporary trends in mesh anchoring techniques. Conversion from laparoscopic to open repair was required in a small proportion of cases, ranging from 5% to 10% across studies, often due to extensive adhesions or intraoperative complications. Smoking history was inconsistently reported but ranged between 9% and 17% among patients undergoing open repair and 11%–14% among those undergoing laparoscopic repair. Some studies also reported hernia size and other patient characteristics, further enriching the dataset (Table 1).

**TABLE 1:** Summary of relevant studies.

Author, year	Country	Sample size	Patients		Age (mean)		BMI (kg/m^2^)		Follow‐up (mean)	
			LR	OR	LR	OR	LR	OR	LR	OR
Carbajo et al. (1999) [23]	Spain	60	30	30	57.8	54.9	Ns	Ns	27	27
Moreno‐Egea et al. (2002) [21]	Spain	22	11	11	60.7	58.6	Ns	Ns	Ns	Ns
Misra et al. (2006) [26]	Insia	66	33	33	45.9	45.2	26.28	25.43	13.7	12.9
Barbaros et al. (2007) [25]	Turkey	46	23	23	50.7	54.1	31.6	31.2	18	20
Olmi et al. (2007) [12]	Italy	170	85	85	60	65	28	28	24	24
Navarra et al. (2007) [22]	Italy	24	12	12	59.3	64.1	Ns	Ns	6	6
Pring et al. (2008) [27]	Australia	58	31	27	64.5	55	Ns	Ns	18	24
Asencio et al. (2009) [24]	Spain	84	45	39	58	60.6	31.35	30.61	12	12
Itani et al. (2010) [5]	USA	146	73	73	61.2	59.6	30.6	31.2	2	2
Eker et al. (2013) [6]	Netherlands	194	94	100	59.1	56.7	28.3	29.3	35	35
Rogmark et al. (2013) [28]	Sweden	133	64	69	58	58	29.3	29.3	2	2
Asencio et al. (2021) [29]	Spain	85	46	39	58	60.3	31.35	30.58	165.7^a^	165.7^a^
Hiekkaranta et al. (2024) [30]	Finland	193	65	128	56.9	Ns	31	Ns	87	Ns
Veenendaal et al. (2023) [31]	Netherlands	88	44	44	60.9^a^	58.03^a^	28.84	28.83	Ns	Ns
Rogmark et al. (2016) [32]	Sweden	133	61	63	Ns	Ns	Ns	Ns	12	12

Abbreviations: LR, laparoscopic repair; Ns, not stated; OR, open repair.

^a^ Median.

### 3.4 Outcomes

#### 3.4.1 Recurrence Rate

**FIGURE 3:**
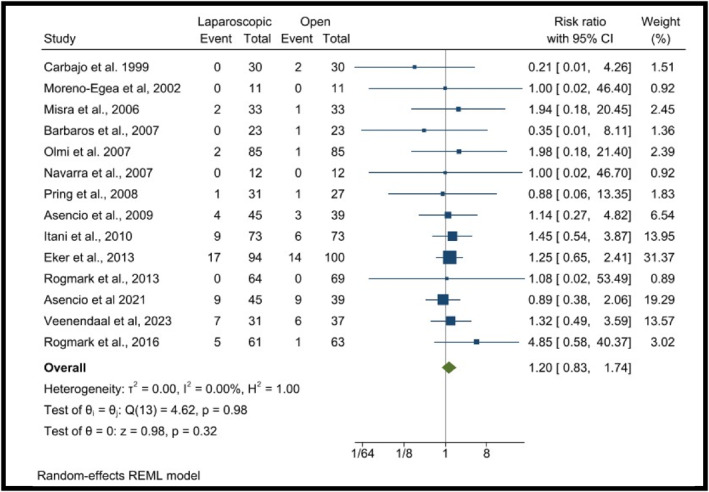
Forest plots showing the recurrence rate.

This analysis included 14 RCTs [5, 6, 12, 21, 22, 23, 24, 25, 26, 27, 28, 29, 31, 32] assessing the recurrence rates of LR and OR incisional hernia repair, analyzing a total of 1502 patients (691 LR, 740 OR). The meta‐analysis compares recurrence rates between LR and OR surgical techniques, yielding an overall RR of 1.20 (95% CI: 0.83–1.74), indicating no significant difference between the two approaches. The *I* ^2^ = 0.00%, suggesting no heterogeneity among the included studies. The Q‐test for heterogeneity (Q (13) = 4.62, *p* = 0.98) confirms the absence of significant variability. The test for the overall effect (*z* = 0.98, *p* = 0.32) shows no statistically significant difference between LR and OR techniques regarding recurrence rates (Figure 3).

#### 3.4.2 Infection

**FIGURE 4:**
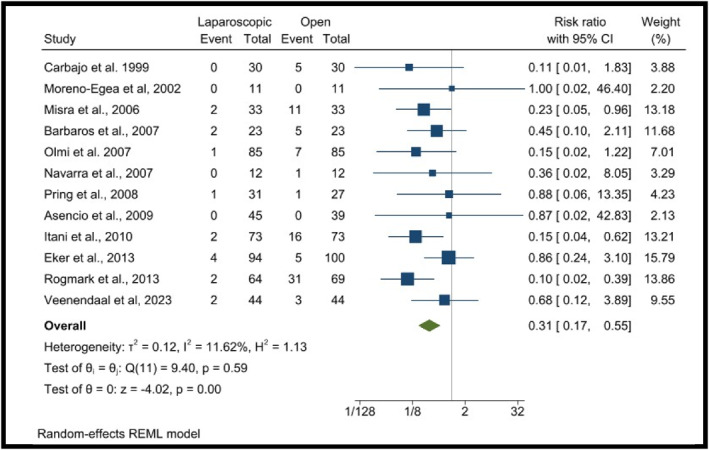
Forest plots showing infection rate.

This meta‐analysis included 12 RCTs [5, 6, 12, 21, 22, 23, 24, 25, 26, 27, 28, 31] assessing the incidence of surgical site infections (SSI) in LR versus OR incisional hernia repair, analyzing a total of 830 patients. The pooled risk ratio was 0.31 [0.17, 0.55], indicating a significantly lower risk of infection in the LR group compared to the OR group. Study‐level findings showed a wide range of risk ratios across studies, with some reporting very low event rates in both groups. The largest contributing study (Eker et al. 2013) accounted for 15.79% of the weight, with a risk ratio of 0.86 [0.24, 1.30]. Heterogeneity analysis demonstrated *I* ^2^ = 11.62%, suggesting low between‐study variability. The overall effect estimates (*p* = 0.00), a highly significant reduction in the risk of surgical site infections in LR compared to OR (Figure 4).

#### 3.4.3 Wound Drainage

**FIGURE 5:**
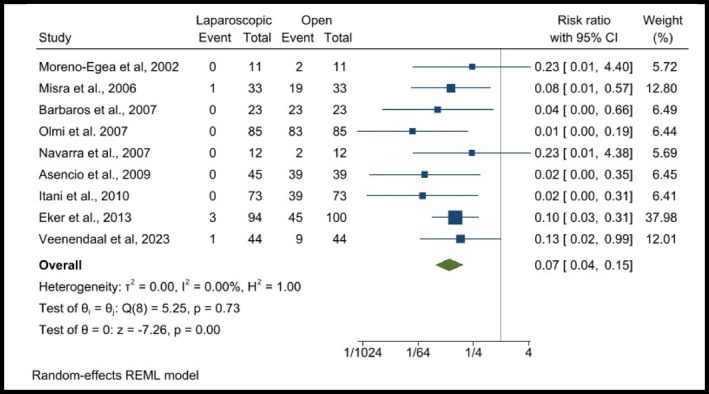
Forest plots showing wound drainage between laparoscopic and open surgery.

Among 8 [5, 6, 12, 21, 22, 24, 25, 26, 31] RCTs with a total of 625 patients. The pooled risk ratio was 0.07 [0.04, 0.15], indicating a significantly lower likelihood of wound drainage in the LR group compared to the OR group. Study‐level findings showed that several trials had zero events in at least one group, leading to wide confidence intervals. The largest contributing study (Eker et al. 2013) accounted for 37.98% of the weight, with a risk ratio of 0.10 [0.03, 0.31]. Heterogeneity analysis demonstrated *I* ^2^ = 0.00%, confirming no significant between‐study variability. The overall effect estimates (*p* = 0.00), a highly significant reduction in the use of wound drainage in LR compared to OR (Figure 5).

#### 3.4.4 Bowel Injury

**FIGURE 6:**
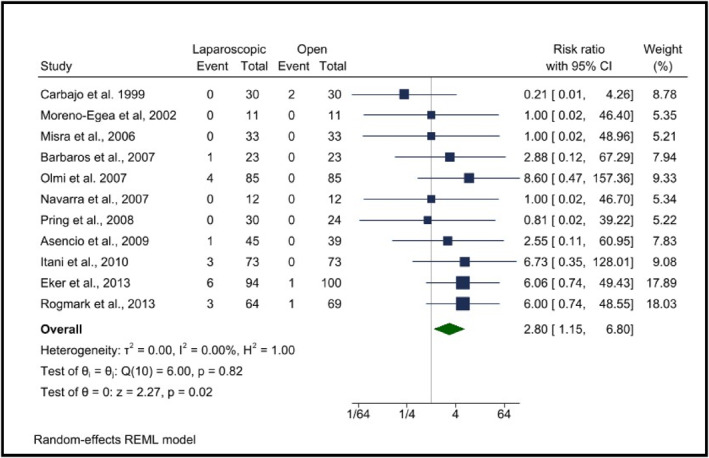
Bowel injury between laparoscopic and open surgery.

The meta‐analysis assessing the risk of bowel injury between LR and OR incisional hernia repair revealed a statistically significant higher risk in the laparoscopic group, with a pooled risk RR of 2.80 (95% CI: 1.15–6.80, *p* = 0.02). This suggests that patients undergoing LR are nearly three times more likely to experience bowel injury compared to OR. Heterogeneity analysis showed *I* ^2^ = 0.00%, *p* = 0.82, indicating minimal variability across studies. The statistical significance (*p* = 0.02) supports the robustness of the findings, with key contributions from studies such as Eker et al. (2013), Itani et al. (2010), and Asencio et al. (2009) (Figure 6).

#### 3.4.5 Publication Bias & Sensitivity Analysis

We assessed publication bias and found no evidence of its presence. The corresponding plots are provided in the supplementary file for reference. Sensitivity analysis was deemed unnecessary, as all significant forest plots exhibited low heterogeneity (*I* ^2^ < 11%), indicating stability and robustness in our finding.

## 4 Discussion

In this updated systematic review and meta‐analysis builds upon the foundational work of Chalabi et al. (2015) [16] by incorporating more recent RCTs and expanding the scope of outcomes analyzed. Laparoscopic incisional abdominal wall hernia repair is a relatively new and evolving technique that can potentially replace open repair. The efficacy and safety of laparoscopic incisional hernia repair is still unclear, as the available evidence comparing the two repair surgical methods is limited. However, the available data from our meta‐analysis nearly all laparoscopic procedures in the included trials were performed using the intraperitoneal onlay mesh (IPOM) technique suggests that laparoscopic repair is as efficient as open repair, if not superior.

Our updated analysis found no significant difference in hernia recurrence rates between LR and OR (RR = 1.20, 95% CI: 0.83–1.74, *p* = 0.32). This suggests that, when performed by experienced surgeons, LR is as durable as OR. This finding aligns with previous systematic reviews and meta‐analyses, which also reported comparable recurrence outcomes between the two approaches, with a pooled *p*‐value of 0.30. Nevertheless, some individual trials have suggested a possible advantage of laparoscopic repair in reducing recurrence [16]. Similarly, a recent systematic review of hybrid intraperitoneal onlay mesh (IPOM) repair for incisional hernias found no statistically significant difference in recurrence rates between hybrid and standard laparoscopic techniques [33]. On the other hand, the evolving surgical landscape now includes advanced techniques like the enhanced view totally extraperitoneal (eTEP) and mini or less open sublay (MILOS) approaches. These techniques are increasingly favored as they allow access to the recommended retrorectus/retromuscular plane for mesh placement while maintaining a minimally invasive, extraperitoneal approach, thereby avoiding the risks associated with intraperitoneal mesh. However, according to the 2023 European Hernia Society (EHS) Guidelines, intraperitoneal mesh placement carries a small but serious risk of bowel injury and adhesions. Therefore, the guidelines recommend minimizing direct mesh contact with intra‐abdominal viscera by placing the mesh in an extraperitoneal plane whenever feasible [11]. Among the included studies, Yang et al. uniquely reported a notable reduction in recurrence with hybrid repair (1.3% vs. 20.5%; *p* < 0.001), specifically in cases involving large defects (> 10 cm) where fascial closure was not achieved laparoscopically [34]. These findings suggest that hybrid repair may offer potential benefits in selected cases of complex or large hernias, where achieving effective fascial closure through a purely laparoscopic approach remains challenging.

Consistent with previous findings, our analysis demonstrates that LR is associated with significantly lower rates of wound infection (RR = 0.19, 95% CI: 0.11–0.32, *p* < 0.00001) and wound drainage (RR = 0.06, 95% CI: 0.03–0.09, *p* < 0.00001) compared to OR. These results align with the minimally invasive nature of laparoscopic surgery, which reduces soft tissue dissection and the need for extensive wound management. The avoidance of large incisions and the reduced exposure of internal tissues to external contaminants likely contribute to these favorable outcomes [6, 35].

However, the risk of bowel injury remains a significant concern in LR. Our updated analysis confirms that the LR approach carries a higher risk of bowel injury compared to OR (RR = 2.80 (95% CI: 1.15–6.80, *p* = 0.02)). This is likely due to the challenges associated with adhesiolysis and the establishment of pneumoperitoneum, which can inadvertently damage intra‐abdominal structures [5]. Surgeons must exercise caution during these steps and be prepared to convert to an open procedure if necessary to mitigate this risk [5, 28]. Although previous meta‐analyses of IPOM repair have not shown significant differences in bowel injury rates between laparoscopic and hybrid approaches, emerging data from focused comparative studies suggest important distinctions in intraoperative safety. In a cohort of 62 patients undergoing either laparoscopic or hybrid intraperitoneal onlay mesh during repair, both groups experienced bowel injuries; however, outcomes varied substantially. In the laparoscopic group, four out of five injuries were missing during the procedure, leading to delayed diagnosis, higher rates of surgical site infection (SSI), and one mortality due to septic shock [26]. Conversely, all injuries in the hybrid group were recognized intraoperatively and managed immediately, resulting in no further complications [22, 26]. These findings suggest that hybrid approaches may offer a safety advantage in complex cases by improving visualization and enabling earlier detection of visceral injuries, thereby potentially reducing morbidity associated with delayed diagnosis.

Our findings demonstrate a statistically significant reduction in the use of wound drainage with the laparoscopic approach compared to open repair (RR = 0.10, 95% CI: 0.03–0.31, *p* = 0.001). This indicates that laparoscopic repair is associated with a substantially lower reliance on postoperative drainage, likely attributable to its minimally invasive nature and reduced tissue trauma. Supporting this observation, a previous meta‐analysis reported that patients who received drainage had a significantly lower incidence of postoperative seroma compared to those without drainage (OR = 0.16, 95% CI: 0.07–0.35, *p* < 0.001) [36]. Similarly, Gao et al. (2020) found a markedly lower incidence of seroma formation in the drainage group compared to the no‐drainage group [37]. Conversely, Sahm et al. (2023) and Willemin et al. (2022) evaluated the outcomes of drainage in the context of open incisional hernia repair. Sahm et al. reported that patients who received drainage were significantly older (63.6 vs. 59.8 years, *p* < 0.001), had higher BMI (29.8 vs. 27.9, *p* < 0.001), elevated ASA scores (*p* < 0.001), larger hernia defects (*p* < 0.001), and more frequent mesh use (92.9% vs. 64.6%, *p* < 0.001) [38]. Willemin et al. observed that the absence of drainage was significantly associated with wound dehiscence (*p* = 0.041), although these events were minor and not linked to an increased risk of surgical site infections [39].

Most of the laparoscopic repairs in our review used the intraperitoneal onlay mesh (IPOM) technique. Therefore, the results related to drainage may not be relevant to newer extraperitoneal laparoscopic methods, such as eTEP or MILOS, which use different surgical planes and techniques. More research is needed to understand drainage outcomes for these modern approaches. Also, these findings underscore the potential importance of selective drain placement in patients undergoing laparoscopic and open incisional hernia repair. However, several potential confounding factors such as smoking status, hernia defect size, mesh position, and suture type were not uniformly reported across the included studies.

## 5 Limitations

Several limitations should be acknowledged in systematic review. First, the laparoscopic data in this review are overwhelmingly representative of the IPOM technique. Second, the limited number of reports included in the studies, such as hospital stay, operation time, and others. Third, recurrence in this review primarily reflects operated recurrence rather than clinical recurrence. As clinical recurrence rates are known to increase over time and are typically higher than operated recurrence, the relatively short follow‐up periods in many included studies may have underestimated the true recurrence rate, introducing an inherent bias. Fourth, some information was not reported in the included studies, such as BMI, mesh type used, smoking status, hernia defect size, mesh position, suture type, and other relevant variables. These unmeasured factors may have influenced postoperative outcomes and should be interpreted as a limitation of the current analysis. Fifth, the risk of bias assessment revealed that some studies had methodological flaws, such as unclear randomization processes or lack of blinding, which could introduce bias into the results. Finally, most included studies were conducted in high‐income countries, which may limit the applicability of our findings to low‐ and middle‐income settings where resources and surgical expertise may differ.

## 6 Conclusion

Laparoscopic repair offers several advantages over open repair, and lower rates of wound infection. However, it is associated with a higher risk of bowel injury, necessitating careful patient selection and surgical expertise. Our findings indicate that, when performed by experienced surgeons, laparoscopic repair is as effective as open repair in preventing hernia recurrence. It's important to note that, majority of the included laparoscopic cases utilized the Intraperitoneal Onlay Mesh (IPOM) technique. The IPOM approach is increasingly disfavored in contemporary practice due to the heightened risk of severe mesh‐related complications, both short‐ and long‐term, arising from visceral contact. Outcomes of hernia repair are also influenced by other key factors, including the surgical approach, the type and position of the mesh used, and the size of the hernia defect. These elements significantly affect recurrence rates, complication risks, and postoperative recovery, as emphasized by recent European Hernia Society (EHS) guidelines. Given that both approaches demonstrate comparable recurrence rates and no significant difference in postoperative pain, the choice of technique should be individualized based on patient characteristics, hernia complexity, and surgeon proficiency. Future studies with longer follow‐up and standardized outcome measures are essential to refine the comparative effectiveness of these techniques, and it should prioritize studies using contemporary, non‐IPOM laparoscopic methods (TAPP/TEP), emphasize on standardized patient‐reported outcomes, cost‐effectiveness, and long‐term complications to guide surgical decision‐making.

## Author Contributions

**Ahmad R. Al‐Qudimat:** conceptualization, data curation, methodology, formal analysis, writing – original draft, writing – review and editing. **Ahmad Zarour:** methodology, writing – review and editing. **Raghad Al‐Taweel:** data curation, writing – original draft. **Amal I. Al‐Awadat:** data curation, writing – original draft. **Hiba Bawadi:** conceptualization, methodology, supervision, validation, writing – review and editing.

## Funding

The authors have nothing to report.

## Ethics Statement

The authors have nothing to report.

## Conflicts of Interest

The authors declare no conflicts of interest.

## Data Availability

All data analyzed during this study are included in this article, and further inquiries can be directed to the corresponding author.
